# Supplementary material for: Evaluation of Different Contrast Agents for Regional Lung Perfusion Measurement Using Electrical Impedance Tomography: An Experimental Pilot Study
Source: J Clin Med. 2023 Apr 7;12(8):2751. doi: 10.3390/jcm12082751 (PMC10143707; doi:10.3390/jcm12082751)
Supplement: Supplementary file 1 [file jcm-12-02751-s001.zip › Caption Figure S1 and video S1-5.pdf]

Caption Figure S1: reproducibility over time

A: signal strength of regional lung perfusion measurements obtained from the maximum amplitude of the regional lung perfusion image after bolus injections of different contrast agents; NaCl: sodium chloride, NaBic: sodium bicarbonate, Glucose: glucose solution, Jonosteril: isotonic balanced crystalloid solution, Iomeprol: non-ionic X-ray contrast agent. Data points show mean values of five animals; dotted lines indicate standard deviations. For each contrast agent, nine repetitive measurements were performed in three sections; the figure shows repetitive measurements in sequential order. A mixed-effects analysis (mixed models for repeated measurements) supports reproducibility over time and showed that differences in signal strength depend on contrast agent but not on time (factor "contrast agent": $p<0.001$; factor "time": $p=0.28$; interaction "contrast agent*time": $p=0.76$).
B-F: figures show individual results of repetitive measurements for single animals and contrast agents; B: NaCl 5.85%, C: NaBic 8.4% D: Glucose 5%, E: Jonosteril, and F: Iomeprol 400mg/mL.

Caption Video S1: EIT-videos of all single NaCl 5.85% injections

EIT-videos of all single NaCl 5.85% injections. Each row represents a single animal, with pig 1 at the top and pig 5 at the bottom. In each row, nine repetitive measurements are displayed in chronological order, with the first injection in the leftmost column and the last injection in the rightmost column. All videos are systematically labelled at the top; the labels include the pig number (t1 to t5), the section number (s1 to s3), and the injection number (inj1 to inj3).

Caption Video S2: EIT-videos of all single NaBic 8.4% injections

EIT-videos of all single NaBic 8.4% injections. Each row represents a single animal, with pig 1 at the top and pig 5 at the bottom. In each row, nine repetitive measurements are displayed in chronological order, with the first injection in the leftmost column and the last injection in the rightmost column. All videos are systematically labelled at the top; the labels include the pig number (t1 to t5), the section number (s1 to s3), and the injection number (inj1 to inj3).

Caption Video S3: EIT-videos of all single Glucose 5% injections

EIT-videos of all single Glucose 5% injections. Each row represents a single animal, with pig 1 at top and pig 5 at bottom. In each row, nine repetitive measurements are displayed in chronological order, with the first injection in the leftmost column and the last injection in the rightmost column. All videos are systematically labelled at the top; the labels include the pig number (t1 to t5), the section number (s1 to s3), and the injection number (inj1 to inj3).

Caption Video S4: EIT-videos of all single Jonosteril injections

EIT-videos of all single Jonosteril injections. Each row represents a single animal, with pig 1 at top and pig 5 at bottom. In each row, nine repetitive measurements are displayed in chronological order, with the first injection in the leftmost column and the last injection in the rightmost column. All videos are systematically labelled at the top; the labels include the pig number (t1 to t5), the section number (s1 to s3), and the injection number (inj1 to inj3).

Caption Video S5: EIT-videos of all single Iomeprol 400mg/mL injections

EIT-videos of all single Iomeprol 400mg/mL injections. Each row represents a single animal, with pig 1 at top and pig 5 at bottom. In each row, nine repetitive measurements are displayed in chronological order, with the first injection in the leftmost column and the last injection in the rightmost column. All videos are systematically labelled at the top; the labels include the pig number (t1 to t5), the section number (s1 to s3), and the injection number (inj1 to inj3).
