# Supplementary figures and images for: Evaluation of Different Contrast Agents for Regional Lung Perfusion Measurement Using Electrical Impedance Tomography: An Experimental Pilot Study
Source: J Clin Med. 2023 Apr 7;12(8):2751. doi: 10.3390/jcm12082751 (PMC10143707; doi:10.3390/jcm12082751)

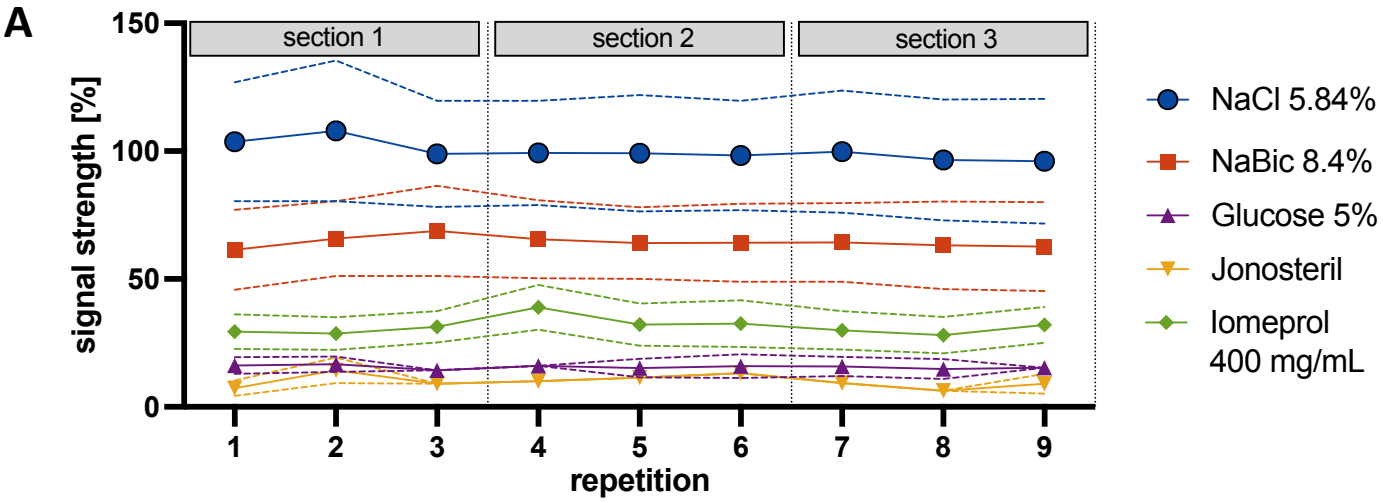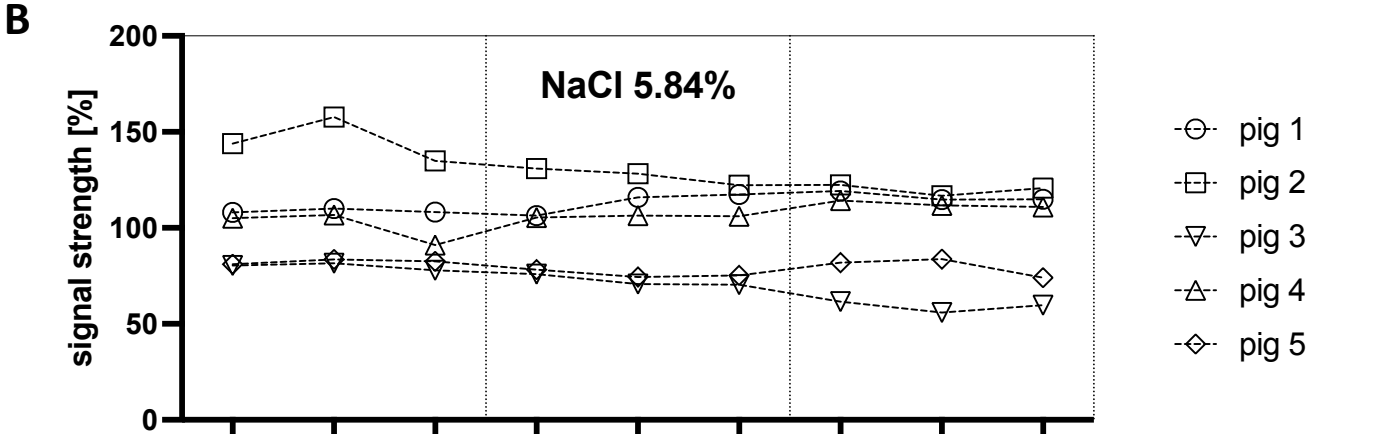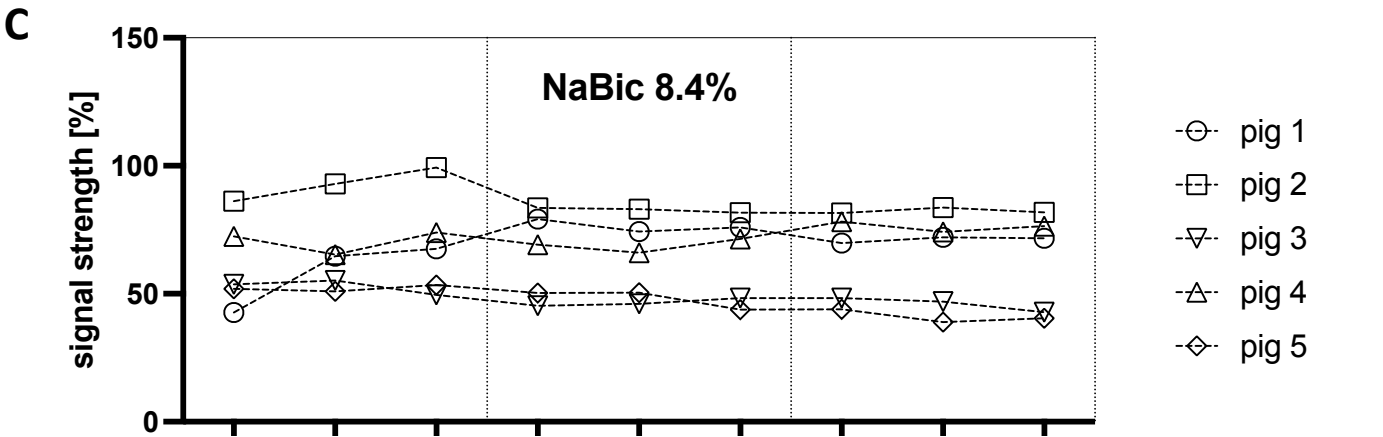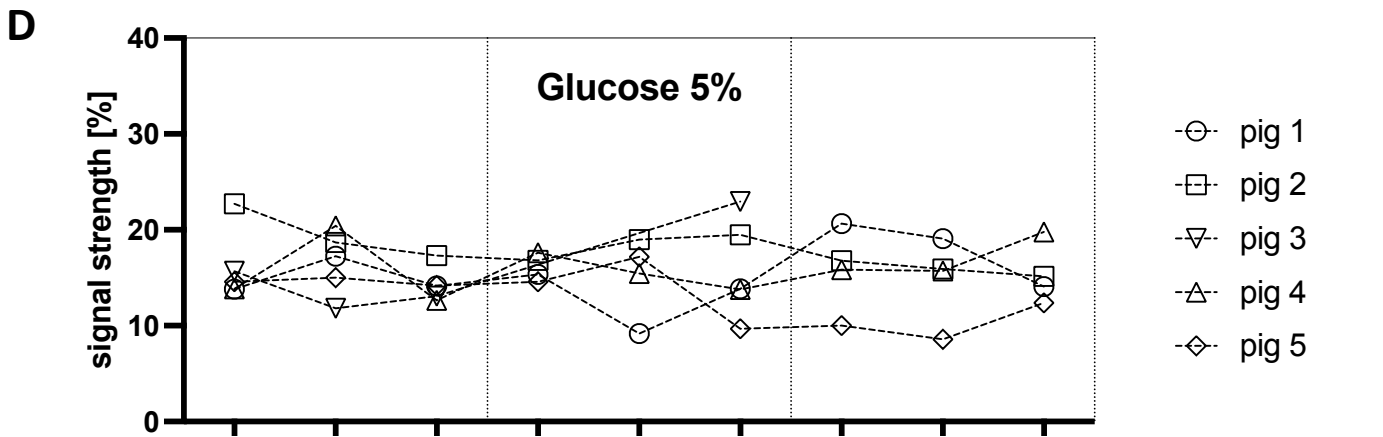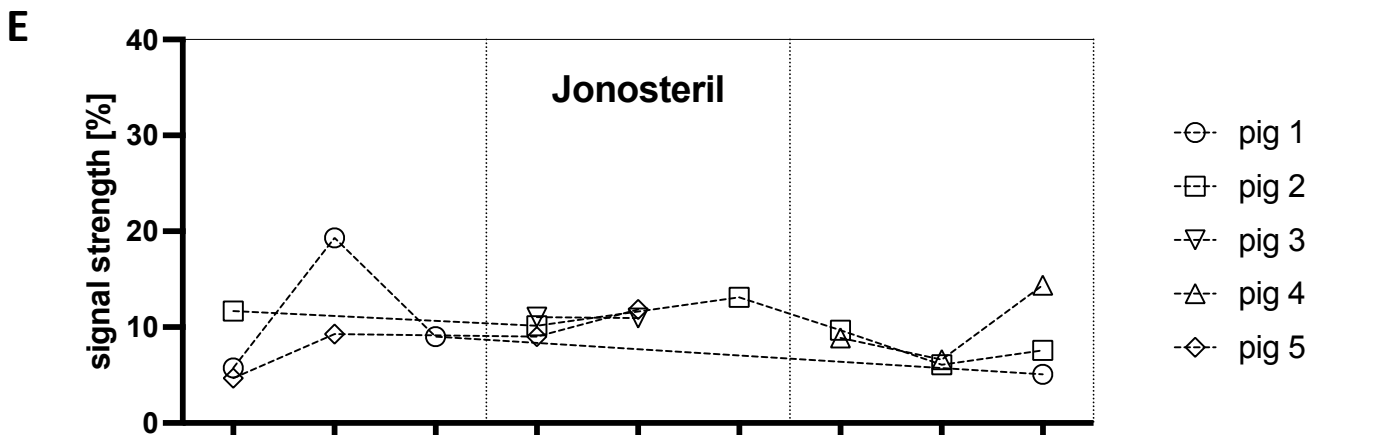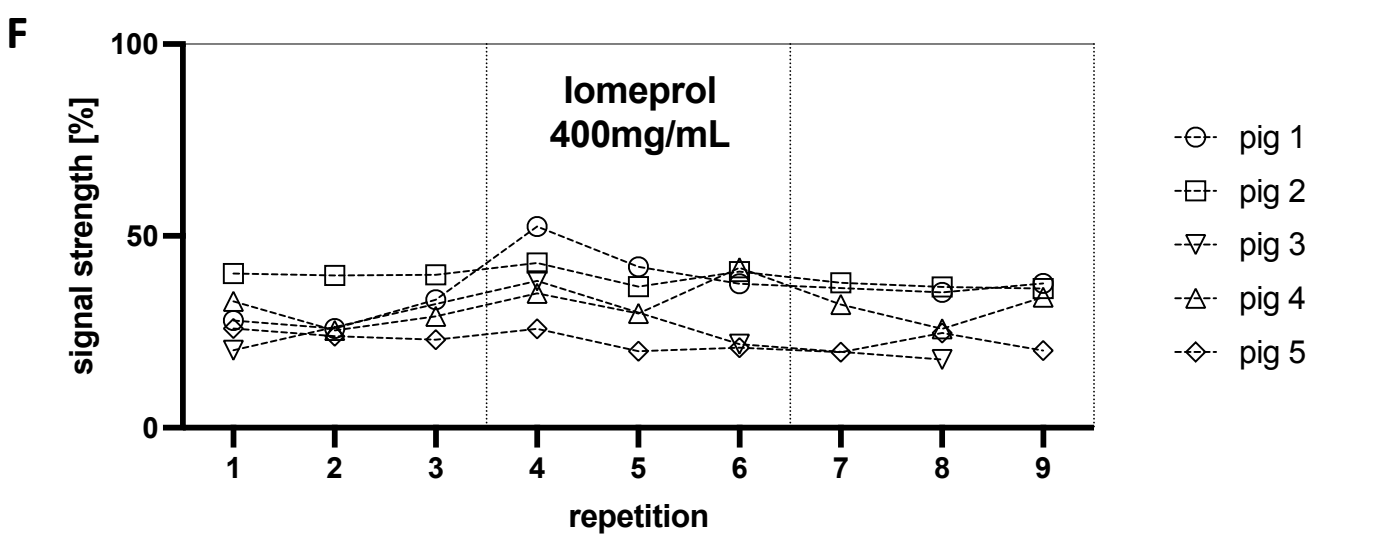

Supplement: Supplementary file 1 [file jcm-12-02751-s001.zip › Muders_Figure-S1.pdf]
